# Differences in acute pain perception between patients and physicians in the emergency department

**DOI:** 10.1016/j.heliyon.2022.e11462

**Published:** 2022-11-10

**Authors:** Malak Alotaibi, Muna Aljahany, Zaid Alhamdan, Mashael Alsaffar, Abdulrahman Almojally, Wajdan Alassaf

**Affiliations:** a Department of Emergency, Prince Mohammad bin Abdulaziz Hospital, Riyadh, Saudi Arabia; b Department of Clinical Sciences, College of Medicine, Princess Nourah Bint Abdulrahman University, Riyadh, Saudi Arabia; c Department of Emergency, King Abdullah bin Abdulaziz Hospital, Princess Nourah bint Abdulrahman University, Riyadh, Saudi Arabia; d Department of Pediatrics, King Faisal Specialist Hospital and Research Center, Riyadh, Saudi Arabia; e Department of Emergency, Ministry of Health, Riyadh, Saudi Arabia

**Keywords:** Pain estimation, Pain score, Emergency department

## Abstract

**Background:**

Pain is a subjective complaint that comprises a vast majority of emergency department (ED)^1^ visits. Owing to its subjectivity, pain reporting is prone to variations that could impact patient care. We aimed to determine the extent of differences in pain rating-scores between patients and their physicians in the ED and impact on patient satisfaction.

**Methods:**

A prospective cross-sectional sample of eligible patients was recruited from two centers in Saudi Arabia. Pain scorings were performed using validated online questionnaires during patients' ED stay.

**Results:**

Pain rating scores by physicians was lower than that by patients (6.3 ± 2.0 versus 7.0 ± 3.1, p = 0.004). Additionally, severe pain rating (8–10 rating) was given less frequently by physicians compared with that by patients (26.0% versus 48.1%, p = 0.004). Comparing the ratings by physicians with those by patients, underestimation was observed in 70.1%, overestimation in 16.9%, and matching rating in 13.0% cases. The most frequent analgesic medication administered was paracetamol (79.2%), followed by diclofenac (26.0%), morphine (10.4%), and ketorolac (9.1%). The medications were administered mainly intravenously (87.0%) and, to a lesser extent, intramuscularly (31.2%). Majority of patients (62.5%) reported not to have sufficient pain relief after treatment.

**Conclusion:**

Most physicians tend to underestimate the level of pain perceived by their patients, which often leads to under-treatment and lower patient satisfaction. The present study revealed a significant difference in pain ratings between patients and physicians.

## Highlights

• There was a significant difference between patient and physician pain ratings
• Pain ratings by physicians were lower than those of patients
• Comparing the physicians' with patients' ratings, underestimation was 70.1%
• Comparing the physicians' with patients' ratings, overestimation was 16.9%,
• Comparing the physicians' with patients' ratings, matching rating was 13.0%.

## 1 Introduction

Pain is one of the most common reasons for visiting the emergency department (ED) [1, 2, 3]. According to the International Association for the Study of Pain, pain is an unpleasant sensation caused by pending or ongoing tissue injury [4]. Pain assessment is a very important step, as it helps in diagnosis, choosing the appropriate analgesic type, monitoring the patient’s condition for improvement or deterioration, and assessing whether the medication is working [5, 6]. There are several reliable and valid methods for pain screening and assessment. One widely used method is the numeric rating scale (NRS), where 0 = no pain and 10 = the worst pain [7]. The NRS is the gold standard for screening, easy to use, and short, and most patients are able to use it [8, 9].

Despite these methods, many published studies [10, 11, 12, 13, 14] have described pain perception differences between patients and their physicians. These studies showed a difference in rating pain by patients and their physicians. In addition, pain is frequently underestimated and undertreated, which may adversely affect the patient’s condition, improvement, and satisfaction [15].

Numerous factors, such as personality type, professional experience, communication, and overcrowding in the ED can influence healthcare providers' pain assessment [16, 17, 18]. These variable factors make pain assessment difficult and inaccurate. In addition, a primary reason for this difference in pain rating is that pain is a subjective and personal experience. Therefore, it is difficult for another person to evaluate a patient’s pain [19]. The Canadian Association of Emergency Physicians consensus document recommends that pain assessment should be performed by patients themselves using pain scales and should not solely rely on the physician’s impression [20].

Although there has been a lot of research on this subject, very few of these have focused on the ED, and there are none in our community. Therefore, the aim of this study was to determine whether there is a significant difference in pain rating scores between patients and their physicians in the ED, and to evaluate patient satisfaction with the pain control before discharge or admission to the ward.

## 2 Methods

### 2.1 Study design and study population

This prospective cross-sectional study was conducted at two hospitals, between September 2020 and October 2021. A convenience sample encompassing 77 patients above 14 years of age who presented to the ED with complaints of acute abdominal, flank, or back pain were invited to participate in the study. Hemodynamically unstable patients, intoxicated patients, pregnant women, and patients with pain of more than 72 h duration, any type of analgesia within the preceding 4 h, and language barriers that can affect NRS understanding were excluded from the study.

### 2.2 Survey tool

Data were obtained using two self-administered online questionnaires. Both questionnaires were developed by the investigators, guided by a literature review [21, 22]. The questionnaires were tested for content validity and reliability in a pilot study. The first questionnaire was designed to be completed by the ED physician. It included two main sections. The first section comprised the physician’s socio-demographic data such as age, gender, language, years of experience, and pain score estimated by the physician without asking the patient about their pain score. The second section of the first questionnaire was related to patient data and included 11 questions: patient’s age, sex, language, location of pain (abdomen, flank, back), duration of pain (acute or chronic [more than 72 h]), and if the patient took analgesics before attending the ED. In addition, it included triage time; first pain medication administered at the time of presentation with dose, route, and time; provisional diagnosis; and disposition time. The second online questionnaire was designed to be completed by the patient at the time of reassessment by the physician, and it included the patient’s pain score based on a 0–10 NRS, before and after the first pain medication and patient satisfaction regarding pain management in general. Pain assessment was done in ED upon initial patients evaluation by the physician and repeated after first pain medication. Patient satisfaction with the treatment was done upon discharge or admission to the ward. The questionnaire was prepared in English and translated into Arabic by a group of experts.

### 2.3 Statistical analysis

Categorical data are presented as frequencies and percentages, whereas continuous data are presented as mean and standard deviation (SD). Differences between physicians' and patients' ratings of the severity of pain perceived by patients were categorized as underestimation, matching rating, or overestimation. Due to the low frequency of matching ratings and overestimations, physicians' and patients' characteristics were compared by physicians' underestimation status (present versus absent). Chi-square or Fisher’s exact test, as appropriate, was used to examine differences in categorical variables, whereas Student’s t-test or Mann Whitney U test, as appropriate, was used to examine differences in continuous variables. Differences in patients' ratings before and after treatment were compared using the Wilcoxon signed-rank test for continuous data and the McNemar test for categorical data. To detect independent predictors of physicians' underestimation, multivariate logistic regression was performed using backward elimination of all variables included in the univariate analysis (Tables 1–3) with a p-value <0.1. All p-values were two-tailed. Statistical significance was set at p < 0.05. SPSS (Version 25.0. Armonk, NY: IBM Corp) was used for all statistical analyses.

**Table 1:** Demographic and professional characteristics of the emergency department physicians by physicians' underestimation of patients' pain (N = 77).

	Physicians' underestimation		Total (N = 77)	p-value
	No (N = 23)	Yes (N = 54)		
**Age (years)**				
Mean ± SD	32.9 ± 7.5	32.6 ± 5.2	32.7 ± 6.0	0.873
<35	15 (65.2%)	35 (64.8%)	50 (64.9%)	0.032
35–44	5 (21.7%)	19 (35.2%)	24 (31.2%)	
≥45	3 (13.0%)	0 (0.0%)	3 (3.9%)	

**Sex**				
Male	13 (56.5%)	41 (75.9%)	54 (70.1%)	0.089
Female	10 (43.5%)	13 (24.1%)	23 (29.9%)	

**Spoken language**				
English	1 (4.3%)	1 (1.9%)	2 (2.6%)	0.511
English and Arabic	22 (95.7%)	53 (98.1%)	75 (97.4%)	
**Years of service in emergency department**				

Mean ± SD	7.3 ± 7.0	7.5 ± 5.0	7.4 ± 5.6	0.392
≤5 years	12 (52.2%)	25 (46.3%)	37 (48.1%)	0.800
6–10 years	7 (30.4%)	16 (29.6%)	23 (29.9%)	
More than 10 years	4 (17.4%)	13 (24.1%)	17 (22.1%)	

**Rating of the severity of pain felt by the patient**				
Mean ± SD	7.2 ± 1.6	5.9 ± 2.0	6.3 ± 2.0	0.006
None (0)	0 (0.0%)	1 (1.9%)	1 (1.3%)	0.016
Mild (1–4)	0 (0.0%)	14 (25.9%)	14 (18.2%)	
Moderate (5–7)	16 (69.6%)	26 (48.1%)	42 (54.5%)	
Severe (8–10)	7 (30.4%)	13 (24.1%)	20 (26.0%)

**Table 2:** Demographic and clinical characteristics of the emergency patients by physicians' underestimation of patients' pain (N = 77).

	Physicians' underestimation		Total (N = 77)	p-value
	No (N = 23)	Yes (N = 54)		
**Age (years)**				
Mean ± SD	32.4 ± 10.3	32.6 ± 11.1	32.5 ± 10.8	0.952
<25	6 (26.1%)	15 (27.8%)	21 (27.3%)	0.904
25–35	8 (34.8%)	16 (29.6%)	24 (31.2%)	
>35	9 (39.1%)	23 (42.6%)	32 (41.6%)	

**Sex**				
Male	11 (50.0%)	20 (37.0%)	31 (40.8%)	0.297
Female	11 (50.0%)	34 (63.0%)	45 (59.2%)	

**Spoken language**				
Arabic	15 (65.2%)	29 (53.7%)	44 (57.1%)	0.148
English	1 (4.3%)	0 (0.0%)	1 (1.3%)	
Both	7 (30.4%)	25 (46.3%)	32 (41.6%)	

**Location of the pain**				
Abdominal pain	13 (56.5%)	36 (66.7%)	49 (63.6%)	0.546
Flank pain	7 (30.4%)	10 (18.5%)	17 (22.1%)	
Back pain	3 (13.0%)	8 (14.8%)	11 (14.3%)	

**Type of complaint**				
Acute (<72 h)	22 (95.7%)	43 (79.6%)	65 (84.4%)	0.095
Chronic (>72 h)	1 (4.3%)	11 (20.4%)	12 (15.6%)	

**Took pain killers before coming to the emergency department**				
No	17 (73.9%)	45 (83.3%)	62 (80.5%)	0.359
Yes	6 (26.1%)	9 (16.7%)	15 (19.5%)	

**If taken pain killers before coming to the emergency department, when?**				
>4 h	2 (33.3%)	4 (44.4%)	6 (40.0%)	>0.99
<4 h	4 (66.7%)	5 (55.6%)	9 (60.0%)	

**Number of analgesic medications administered at the emergency department**				
Mean ± SD	1.3 ± 0.4	1.3 ± 0.7	1.3 ± 0.6	0.724
Single	17 (73.9%)	36 (69.2%)	53 (70.7%)	0.681
Multiple	6 (26.1%)	16 (30.8%)	22 (29.3%)	

**Types of analgesic medications**				
Paracetamol (1000 mg)	16 (69.6%)	45 (83.3%)	61 (79.2%)	0.222
Diclofenac (75 mg)	7 (30.4%)	13 (24.1%)	20 (26.0%)	0.560
Morphine (3–5 mg)	2 (8.7%)	6 (11.1%)	8 (10.4%)	>0.99
Ketorolac (15–30 mg)	3 (13.0%)	4 (7.4%)	7 (9.1%)	0.420
Fentanyl (0.05–0.10 mg/mL)	1 (4.3%)	2 (3.7%)	3 (3.9%)	>0.99
Ketamine (12.5 mg)	0 (0.0%)	1 (1.9%)	1 (1.3%)	>0.99

**Other medications administered at the emergency department**				
Any	4 (17.4%)	6 (11.1%)	10 (13.0%)	0.474
Omeprazole	2 (8.7%)	4 (7.4%)	6 (7.8%)	>0.99
Metoclopramide	1 (4.3%)	0 (0.0%)	1 (1.3%)	0.299
Ondansetron	1 (4.3%)	0 (0.0%)	1 (1.3%)	0.299
Butylbromide	0 (0.0%)	2 (3.7%)	2 (2.6%)	>0.99
Diphenhydramine	1 (4.3%)	0 (0.0%)	1 (1.3%)	0.299

**Route**				
Intravenous	17 (73.9%)	50 (92.6%)	67 (87.0%)	0.057
Intramuscular	9 (39.1%)	15 (27.8%)	24 (31.2%)	0.325

**Duration of stay in emergency department (hours: minutes)**				
Before starting pain medication	0:18 ± 0:20	0:260:39	0:24 ± 0:35	0.500
During pain medication	2:50 ± 2:54	2:52 ± 2:56	2:52 ± 2:54	0.871
Total length of stay	3:10 ± 2:55	3:19 ± 3:01	3:16 ± 2:58	0.782

**Table 3:** Pain questionnaire among emergency patients by physicians' underestimation of patients' pain (N = 77).

	Physicians' underestimation		Total (N = 77)	p-value
	No (N = 23)	Yes (N = 54)		
**Were you in pain during your visit to the emergency department?**				
No	0 (0.0%)	0 (0.0%)	0 (0.0%)	NA
Yes	23 (100.0%)	54 (100.0%)	77 (100.0%)	

**Was your pain unbearable?**				
No	2 (8.7%)	5 (9.3%)	7 (9.1%)	>0.99
Yes	21 (91.3%)	49 (90.7%)	70 (90.9%)	

**Were you offered pain-relieving medication during your admission?**				

No	0 (0.0%)	0 (0.0%)	0 (0.0%)	NA
Yes	23 (100.0%)	54 (100.0%)	77 (100.0%)	

**If your answer is (yes), was the treatment sufficient to relieve the pain?**				

No	1 (4.3%)	7 (13.0%)	8 (10.4%)	0.423
Yes	22 (95.7%)	47 (87.0%)	69 (89.6%)	

**If your answer is (No), do you think you should have received other treatments?**				

No	0 (0.0%)	0 (0.0%)	0 (0.0%)	>0.99
Yes	1 (100.0%)	4 (57.1%)	5 (62.5%)	
Do not know	0 (0.0%)	3 (42.9%)	3 (37.5%)	

**On a scale from 0 (no pain) to 10 (most painful), how would you rate the severity of your pain**				

After treatment	5.4 ± 3.9	1.3 ± 1.7	2.6 ± 3.1	<0.001
Before treatment	4.1 ± 3.7	8.2 ± 1.7	7.0 ± 3.1	<0.001

**Difference in rating after treatment compared with before treatment**				

Mean ± SD	1.3 ± 7.4	-6.8 ± 1.9	-4.4 ± 5.7	<0.001
Improvement	10 (43.5%)	54 (100.0%)	64 (83.1%)	<0.001
No change	1 (4.3%)	0 (0.0%)	1 (1.3%)	
Worsened	12 (52.2%)	0 (0.0%)	12 (15.6%)	

**Overall, how would you rate the care you received today?**				

Weak	0 (0.0%)	0 (0.0%)	0 (0.0%)	0.782
Medium	0 (0.0%)	1 (1.9%)	1 (1.3%)	
Good	2 (8.7%)	2 (3.7%)	4 (5.2%)	
Very good	6 (26.1%)	15 (27.8%)	21 (27.3%)	
Excellent	15 (65.2%)	36 (66.7%)	51 (66.2%)	

### 2.4 Ethical considerations

Ethical approval was obtained from the Institutional Review Board of Princess Nourah Bint Abdulrahman University (IRB log number 20-0132). Both the physicians and patients who agreed to participate provided written informed consent.

## 3 Results

**Figure 1:**
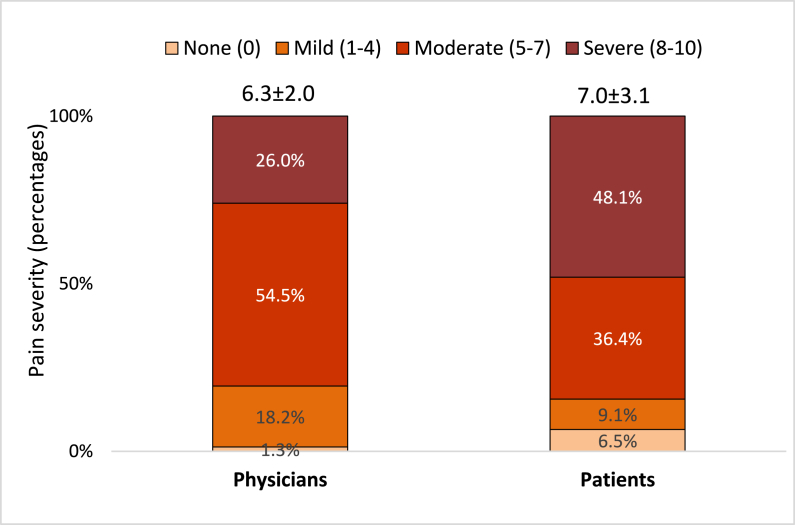
Difference between physicians' and patients' rating of the severity of pain perceived by patients (n = 77) Note: p-value of Mann-Whitney test was 0.004 for continuous data and p-value of Fisher exact test was 0.004 for categorical data.

As shown in Figure 1, pain rating scores by physicians were lower than that by patients (6.3 ± 2.0 versus 7.0 ± 3.1, p = 0.004). Additionally, severe pain rating (8–10 rating) was given less frequently by physicians compared with that by patients (26.0% versus 48.1%, p = 0.004). Comparing the ratings by physicians with those by patients, underestimation was observed in 70.1%, overestimation in 16.9%, and matching rating in 13.0% cases.

Table 1 shows the demographic and professional characteristics of the physicians who showed underestimation of the severity of patients' pain. The average age of the physicians was 32.7 ± 6.0 years with the majority (64.9%) under 35 years. Majority of the physicians were male (70.1%) and spoke both Arabic and English (97.4%). Their average duration of service in the ED was 7.4 ± 5.6 years (48.1% with ≤5 years and 22.1% with >10 years). The average rating of the severity of pain by physicians was 6.3 ± 2.0. As per their rating, the most frequent severity was moderate pain (54.5%), followed by severe (26.0%), and lastly mild (18.2%). Physicians' underestimation of patients' pain was significantly associated with age <35 years (p = 0.032), lower physicians' pain rating scores (p = 0.006). Additionally, there was a trend toward association of physicians' underestimation of patients' pain with male sex (p = 0.089).

Table 2 shows demographic and clinical characteristics of the ED patients. The average age was 32.5 ± 10.8 years. Majority of the patients were females (59.2%) and spoke only Arabic (57.1%) or both English and Arabic (41.6%). The most frequent complaint was abdominal pain (63.6%) and most complaints were of acute pain (84.4%). Only 19.5% of the patients had taken pain killers before coming to the ED mainly within 4 h (60.0%). Majority (70.7%) of the patients were treated with a single analgesic medication at the ED, whereas 29.3% patients were treated with multiple analgesic medications (average 2.1 ± 0.5 medications). The most frequent analgesic medication administered was paracetamol (79.2%), followed by diclofenac (26.0%), morphine (10.4%), and ketorolac (9.1%). Additional medications were administered for 13.0% of the patients; the most frequent was omeprazole (7.8%). The medications were administered mainly intravenously (87.0%) and to lesser extent, intramuscularly (31.2%). The medications were administered after an average of 24 ± 35 min after admission and patients spent an average of 3 h and 16 min in the ED. None of the above patients' characteristics were significantly associated with physicians' underestimation of patients' pain. Nevertheless, there were trends of association between physicians' underestimation and chronic complaints (p = 0.095) and intravenous route (p = 0.057).

**Figure 2:**
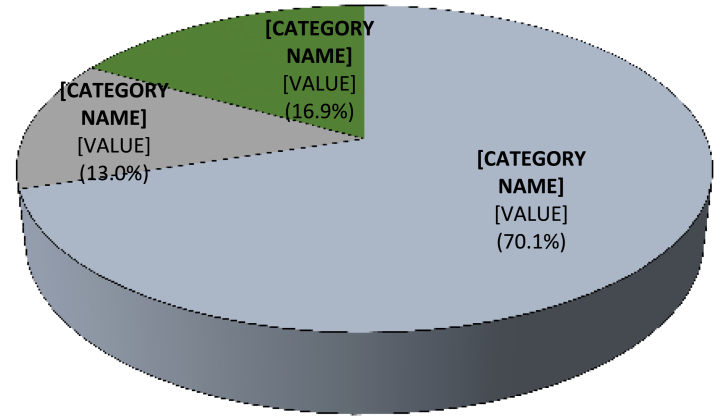
Difference between patients' rating of the severity of pain before and after receiving treatment (n = 77) Note: p-value of Wilcoxon signed ranks test was <0.001 for continuous data and p-value of McNemar test was <0.001 for categorical data.

**Figure 3:**
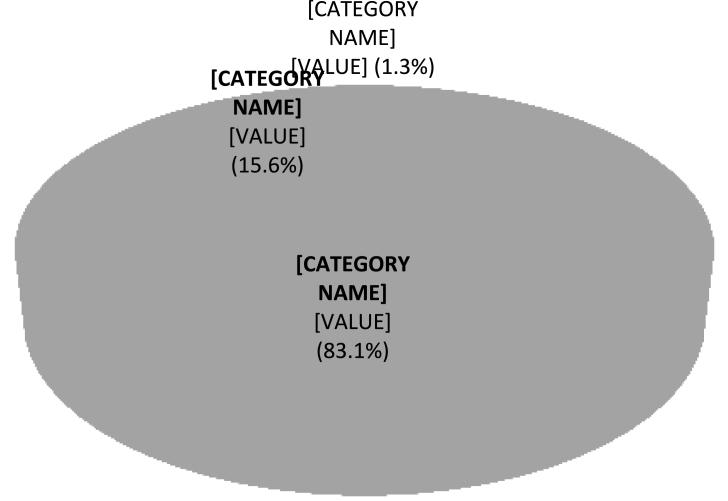
Percentages of improvement, worsening, and no change among patients related to severity of pain.

Table 3 shows the observations from the pain questionnaire among emergency patients. All patients had pain on admission, which was largely unbearable (90.9%). All patients were offered analgesic medication(s), which was (were) mostly sufficient to relieve the pain (89.6%). The majority (62.5%) of patients who did not get sufficient pain relief felt they should have received other treatments. The majority (83.1%) of patients who experienced pain relief gave much lower pain rating after treatment than that before treatment (2.6 ± 3.1 versus 7.0 ± 3.1, p < 0.001). The pain rating reduction was 4.4 ± 5.7 points in all patients and 6.8 ± 1.9 in those who experienced improvement. As shown in Figure 2, severe pain was reduced from 48.1% to 13.0% and moderate pain was reduced from 36.4% to 7.8% (p < 0.001). Majority of the patients rated the care received at the ED as excellent (66.2%), followed by very good (27.3%). Of all the questionnaire questions, patients' pain rating before and after treatment and pain improvement were significantly associated with the physicians' underestimation of patients' pain (p < 0.001 for all) (see Figure. 3).

Table 4 shows the multivariate logistic regression analysis of the potential predictors of physicians' underestimation of patients' pain. After adjusting for the variables that were associated significantly (p < 0.05) or marginally (p < 0.1) with underestimation in univariate analysis (Tables 1–3), one-unit decrease (improvement) in patients' rating (of pain) after treatment was associated with 82% increase in the odds of underestimation (p = 0.005). Additionally, one-unit higher pain rating by physicians at admission was associated with 66% decrease in the odds of underestimation (p = 0.002).

**Table 4:** Multivariate∗ logistic regression analysis of potential predictors of physicians' underestimation of patients' pain.

	Odds ratio (OR)	Confidence intervals of OR	p-value
Difference in patients' rating after treatment compared with before treatment (improvement)	1.82	1.20–2.77	0.005
Physicians' rating of the severity of pain perceived by the patient at admission	0.33	0.17–0.66	0.002

Adjusted for physicians' age and sex, as well as patients' type of complaint and intravenous route. R-square 0.75.

## 4 Discussion

Multivariate analysis showed that none of patient and physician characteristics were independently associated with physicians' underestimation of patients' pain. While patients tend to overestimate their pain, physicians' rating was probably more objective than patients' rating as all patients whose rating was underestimated by physicians experienced improvement and underestimation was significantly associated with higher degree of improvement.

Since pain is a personal experience; it must be managed according to a set of objective principles [21]. Doctors and nurses do not appropriately address acute pain despite evidence-based recommendations [22]. Inadequate pain assessment and lack of information about pain are two of the most common impediments for healthcare practitioners [23].

Patients complaining of pain account for 60–70% of all visits to the ED [24]. The present study showed that majority of the patients were women (59.2%). In contrast, Baharuddin et al. [25] reported that majority of the patients who visited the ED for pain were men, with no significant differences in the pain severity scores of male and female patients; a greater proportion of female patients reported severe pain, and none reported mild pain. These results are congruent with existing human studies on gender differences in the experience of pain, which show that women are more sensitive to pain than men [26]. However, a study among people in Singapore revealed that sex did not influence the median pain score [27].

The present study showed the most frequent severity reported by patients were moderate pain (54.5%), followed by severe (26.0%) and mild (18.2%). However, according to a study done by Baharuddin et al. [25] 44.8 % of patients experienced moderate pain, whereas none of them experienced severe pain. The present study revealed a significant difference between the patient and physician pain ratings. The pain rating by physicians (6.3 ± 2.0) was lower than that by patients (7.0 ± 3.1). Additionally, severe pain rating was given less frequently by physicians (26%) compared with that by patients (48.1%; p = 0.004). Comparing the ratings by physicians with those by patients, underestimation was observed in 70.1% cases.

The average rating of the severity of pain by physicians was 6.3 ± 2.0. A study by Kamarul et al. [25] reported an average score of 5.6 ± 1.8. The average patient rating of pain severity in our study was 7.0 ± 3.1. Dale J et al. [28] reported an average pain score of 4.9, which is somewhat lower than that of the present study; however, a study conducted by Todd et al. reported a higher intensity pain score [29]. The most frequent analgesic medication administered was paracetamol (79.2%), followed by diclofenac (26.0%), morphine (10.4%), and ketorolac (9.1%) in present study. However, Todd KH et al. [29] reported that majority of the analgesics administered were opioids (59%); morphine was the most used analgesic (20%), followed by ibuprofen (17%).

This study has some limitations. Increasing the number and variability of patients and the background of treating physicians would have made the results more generalizable. The presumed patient and physician factors leading to pain underestimation were not proven in this study (e.g., being a female patient, less experienced physician). To overcome this phenomenon, we suggest giving more emphasis on pain estimation and management in medical school and residency training, utilizing a standardized approach based on frequent assessment and re-assessment of unified modules, and promoting a more empathetic approach to patients presenting with pain among healthcare providers.

## 5 Conclusion

Most physicians tend to underestimate the level of pain perceived by their patients, which often leads to under-treatment and lower patient satisfaction. The present study showed a significant difference in pain ratings between patients and physicians. There were also substantial disparities in the mean pain scores of patients upon arrival compared to those of physicians. Consequently, requesting pain ratings is a crucial step towards effective pain management in emergency medicine.

## Declarations

### Author contribution statement

Malak Alotaibi: Conceived and designed the experiments; Performed the experiments; Wrote the paper.

Muna Aljahany: Conceived and designed the experiments; Performed the experiments; Contributed reagents, materials, analysis tools or data; Wrote the paper.

Zaid Alhamdan, Mashael Alsaffar, Abdulrahman Almojally and Wajdan Alassaf: Contributed reagents, materials, analysis tools or data; Wrote the paper.

### Funding statement

The authors are grateful to Princess Nourah bint Abdulrahman University for supporting this research through sabbatical leave program.

### Data availability statement

Data will be made available on request.

### Declaration of interest’s statement

The authors declare no conflict of interest.

### Additional information

No additional information is available for this paper.

## Appendix A Supplementary data

Data Collection Form Data Collection Form
