# Supplementary material for: Differences in acute pain perception between patients and physicians in the emergency department
Source: Heliyon. 2022 Nov 10;8(11):e11462. doi: 10.1016/j.heliyon.2022.e11462 (PMC9667246; doi:10.1016/j.heliyon.2022.e11462)
Supplement: Data Collection Form [file mmc1.pdf]

Emergency Health Care Providers (EHCP) Data Collection Form
Evaluation of the patient's pain in the emergency room

1. What is your age?
   - ○ 25-34
   - ○ 35-44
   - ○ 45-54
   - ○ 55-64
   - ○ 65-74

2. What is your sex?
   - ○ Male.
   - ○ Female.

3. Which language do you speak?
   - ○ Arabic.
   - ○ English.
   - ○ Both.

4. What is your job title?
   - ○ Physician.
   - ○ Nurse.

5. Years of service in the emergency department:
   - ○ 5 years or less.
   - ○ 6-10 years.
   - ○ 11-15 years.
   - ○ More than 15 years.

6. On a scale from 0 to 10, how do you rate the intensity of pain felt by the patient?
   ....

Patient Data Collection Form
Evaluation of the patient's pain in the emergency room

1. MRN: ....

2. Patient's age:
   ....

3. Patient's sex:
   ○ Male.
   ○ Female.

4. Patient's language:
   ○ Arabic.
   ○ English.
   ○ Both.

5. Location of the patient's pain:
   ○ Abdominal pain.
   ○ Flank pain.
   ○ Back pain.

6. Duration of patient's complaint:
   ○ Acute.
   ○ Chronic (> 72 hrs).

7. Did the patient take painkillers before coming to the ER?
   ○ Yes,
   ↳ When: ○ More than 4 hours.
   ○ Less than 4 hours.
   ○ No.

8. Triage time: _______:_______

9. First pain medication given at the time of presentation with dose and route:
   .... Dose: .... Route: ....

10. Time of first pain medication: _____:______

11. Provisional diagnosis:
    ………………………………………………………………

12. Disposition time: _______:_______
